# Marked Genome Reduction Driven by a Parasitic Lifestyle: Two Complete Genomes of Endosymbiotic Bacteria Possibly Hosted by a Dinoflagellate

**DOI:** 10.1264/jsme2.ME25005

**Published:** 2025-06-05

**Authors:** Takuro Nakayama, Ryo Harada, Akinori Yabuki, Mami Nomura, Kogiku Shiba, Kazuo Inaba, Yuji Inagaki

**Affiliations:** 1 Center for Computational Sciences, University of Tsukuba, Tsukuba, Ibaraki, Japan; 2 Faculty of Medicine, Dalhousie University, Halifax, Nova Scotia, Canada; 3 Research Institute for Global Change (RIGC), Japan Agency for Marine-Earth Science and Technology (JAMSTEC), Yokosuka, Kanagawa, Japan; 4 Advanced Institute for Marine Ecosystem Change (WPI-AIMEC), Yokohama, Kanagawa, Japan; 5 Faculty of Science, Yamagata University, Yamagata, Yamagata, Japan; 6 Shimoda Marine Research Center, University of Tsukuba, Shimoda, Shizuoka, Japan

**Keywords:** *Gammaproteobacteria*, *Fastidiosibacteraceae*, phylogenomics, genome reduction, parasitism

## Abstract

Bacteria with endosymbiotic lifestyles often show marked genome reduction. While the shrinkage of genomes in intracellular symbionts of animals, including parasitic bacteria, has been extensively exami­ned, less is known about symbiotic bacteria associated with single-celled eukaryotes. We herein report the genomes of two novel gammaproteobacterial lineages, RS3 and XS4, identified as putative parasitic endosymbionts of the dinoflagellate *Citharistes regius*. Phylogenetic ana­lyses suggest that RS3 and XS4 belong to the family *Fastidiosibacteraceae* within the order *Beggiatoales*, forming independent lineages therein. The genomes of RS3 and XS4 are 529 and 436‍ ‍kbp in size, respectively, revealing marked reductions from related bacterial genomes. XS4, which has a very reduced genome with a low GC content, uses a different genetic code, in which UGA assigned tryptophan. The small genomes of RS3 and XS4 encode a limited number of proteins, retaining only approximately 20% of the predicted ancestral proteome. Metabolic reconstruction suggests that RS3 and XS4 are parasitic symbionts that are heavily dependent on their host for essential metabolites. Furthermore, we found that the ancestor of both genomes likely acquired an ADP:ATP antiporter gene via horizontal gene transfer, an event that may have enabled their evolution as energy parasites by facilitating the acquisition of ATP from their host. These results on novel bacteria with highly reduced genomes expand our understanding of the phylogenetic and genomic diversities of endosymbiotic bacteria in protists.

In natural environments, many bacteria establish symbiotic relationships with various eukaryotic organisms, including mutualistic, commensalistic, and parasitic interactions. The intracellular bacterial symbionts of arthropods have been extensively exami­ned. They provide metabolic complementation by supplying substances that the host cannot synthesize (*e.g.*, specific amino acids) (McCutcheon and Moran, 2011), confer defensive capabilities against host predators (Oliver *et al.*, 2003), or, in some cases, manipulate host reproductive systems to facilitate their own transmission to the next generation (Katsuma *et al.*, 2022). These symbiotic relationships have a significant impact on the evolution of these bacteria, particularly their genomes. Bacteria that form intimate symbiotic relationships with eukaryotic hosts frequently undergo genome reduction, shedding genes that are unnecessary for their specialized lifestyle over their evolution. This may result in extremely small genomes, sometimes less than 1‍ ‍Mbp in size (McCutcheon and Moran, 2011). Investigation of these minimal genomes is crucial, not only to elucidate the specific mechanisms underlying each symbiosis, but also to identify the essential gene sets required for bacterial survival (Koonin, 2003), which will provide valuable insights into fundamental biological processes.

To gain a comprehensive understanding of genome reduction in symbiotic bacteria, it is essential to investigate various symbiotic relationships hosted by a wide range of phylogenetic groups of eukaryotes. While previous studies revealed that phylogenetically diverse bacteria engage in symbiotic relationships with eukaryotes and analyzed their genomes (Moran *et al.*, 2008; McCutcheon and Moran, 2011), most of these studies concentrated on symbiotic bacteria associated with animal hosts. However, advances in mole­cular phylogenetics have demonstrated that multicellular organisms, including animals, represent only a small fraction of overall eukaryotic diversity (Burki *et al.*, 2020). The majority comprises single-celled eukaryotes, or protists, and these organisms frequently harbor symbiotic bacteria (Nowack and Melkonian, 2010; Husnik *et al.*, 2021). Nevertheless, the phylogenetic and genomic diversities of symbiotic bacteria in protists have not yet been exami­ned as extensively as those in animals (Husnik *et al.*, 2021).

Dinoflagellates, belonging to *Alveolata*, represent a prominent group of protists thriving in various aquatic environments. These organisms are very biodiverse, with some lineages known to have symbiotic bacteria (Gavelis and Gile, 2018; Foster and Zehr, 2019; Husnik *et al.*, 2021); however, the genomic-level characterization of these bacterial symbionts warrants further study. Genome ana­lyses have recently been conducted on cyanobacteria symbiotic with marine *Dinophysales* dinoflagellates (Nakayama *et al.*,‍ ‍2019, 2024). Previous studies suggested the presence of‍ ‍additional, non-cyanobacterial symbionts within *Dinophysales*, including members of *Gammaproteobacteria*, *Alphaproteobacteria*, and *Betaproteobacteria* (Foster *et al.*, 2006; Farnelid *et al.*, 2010). However, only partial sequences of the *nifH* and 16S rRNA genes have been obtained, which has left their metabolic characteristics largely unknown.

A recent study focused on and sequenced the genome of a cyanobacterial episymbiont of the *Dinophysales* dinoflagellate *Citharistes regius* (Nakayama *et al.*, 2024). The cyanobacterial genome was recovered from a genome assembly generated from the amplified DNA of a whole *C. regius* cell. Our re-ana­lysis of this assembly also revealed additional genomic sequences representing putative endosymbiotic bacteria. We herein report on these genomic sequences and discuss the inferred evolutionary and metabolic characteristics of these bacteria. The results obtained herein revealed that these bacteria, designated as RS3 and XS4, have undergone extreme genome reduction, likely as an adaptation to their putative intracellular lifestyle and intimate association with the host dinoflagellate. They exhibit features of nutritional parasitism, relying heavily on their host for essential metabolites. Additionally, XS4 displays a unique genetic code alteration, further highlighting the evolutionary changes associated with its reduced genome and parasitic lifestyle.

## Materials and Methods

### Single-cell genome amplification, sequencing, and assembly

*C. regius* sampling, whole-genome amplification, and genome assembly were performed as described by Nakayama *et al.* (2024). Briefly, a cell of *C. regius* (isolate M16) was found in a seawater sample collected with a plankton net off Shimoda, Shizuoka Prefecture, Japan (N34°29.222′, E139°06.200′). The cell was collected using a glass microcapillary and washed four times: three times by transferring it through droplets of sterilized seawater and then once with PCR-grade sterilized water, to remove residual seawater before placing it in sterilized fresh water for subsequent whole-genome amplification. Sterilized seawater was prepared by filtering natural seawater collected off the Ogasawara Islands, Japan, through a glass microfiber filter with a nominal pore size of 0.7‍ ‍μm (Whatman GF/F), followed by autoclaving at 121°C for 20‍ ‍min. Whole-genome amplification was performed using the REPLI-g Single Cell Kit (QIAGEN), and the amplified DNA sample was treated with S1 nuclease (TaKaRa) to reduce branching junctions. Amplified DNA was analyzed using Illumina NovaSeq 6000 and Oxford Nanopore MinION. The genome was assembled *de novo* in a hybrid manner, incorporating both Illumina short reads and nanopore long reads, using Unicycler (version 0.4.8) with the --no_pilon option as described by Nakayama *et al.* (2024). A preliminary annotation of the hybrid assembly of the amplified genomes was generated using the DFAST web service (Tanizawa *et al.*, 2016, 2018). Predicted 16S rRNA gene sequences in the assembly were extracted and subsequently analyzed using the NCBI BLAST search service to examine their taxonomic origins and identify potential non-cyanobacterial genomes.

### Estimation of genetic codes and genome annotations

The preliminary genome annotation using the DFAST web service with default options revealed locations in the XS4 genome where open reading frames encoding proteins that are typically conserved in bacteria were interrupted by UGA, one of the stop codons in the standard genetic code. Since XS4 may utilize a different genetic code to that of typical bacteria, we estimated the genetic codes of RS3 and XS4 by focusing on highly conserved amino acid (HCAA) positions within protein sequences conserved across bacterial lineages. We exami­ned the codons used to encode these amino acids in the RS3 and XS4 genomes as follows. We used the tBLASTn program to search the RS3 and XS4 genomes for regions likely to encode highly conserved, single-copy protein-coding genes. Sequence similarity searches of rRNA genes obtained from the preliminary annotation suggested that RS3 and XS4 are both closely related to *Fastidiosibacter lacustris* (genome accession number: GCF_003428155.1) among the bacterial genomes included in the Genome Taxonomy Database (GTDB) (Parks *et al.*, 2022) r214. Therefore, we used the protein sequences of this genome as queries for tBLASTn. The target protein genes were 105 of the 120 genes encoding proteins used as phylogenetic markers in GTDB (bac120), detected from at least one of RS3 and XS4 (Supplementary Table S1). In parallel, we retrieved the aforementioned 105 proteins from 198 bacterial genome sequences representing diverse lineages included in GTDB r214 (Supplementary Table S1). Each set of 105 homologous protein sequences from the 198 bacteria was aligned using the L-INS-i method in MAFFT (version 7.490) (Katoh and Standley, 2013) to align evolutionarily homologous positions, resulting in 105 protein multiple alignments. Based on the amino acid positions in these alignments, we identified positions where 90% or more of the bacterial sequences had the same amino acids. We assumed that these HCAA were also utilized at the corresponding positions in the orthologous proteins of RS3 and XS4. Based on this assumption, we searched for sites corresponding to the HCAA positions within the homologous protein-coding sequences of the RS3 and XS4 genomes detected by tBLASTn, and investigated the codons used to encode these amino acids in each genome. The number of HCAA positions available for genetic code predictions in RS3 and XS4 were 10,707 and 10,214 sites, respectively. We counted the combinations of amino acid types at HCAA positions and the codons found in the RS3 and XS4 genomes, generating a 64×20 count matrix. This matrix was input into Weblogo3 (https://weblogo.threeplusone.com/create.cgi) (Crooks *et al.*, 2004) to create sequence logos representing amino acid frequencies at HCAA positions corresponding to each codon. The Weblogo3 options used were as follows: Units, probability; Composition, No adjustment for composition.

The genome annotation of RS3 and XS4 was performed using the DFAST web service (version 1.6.0) (Tanizawa *et al.*, 2016, 2018). Analyses of the genetic code suggested that the proteins of the RS3 genome are encoded by the standard bacterial genetic code (NCBI’s translation table 11), while the proteins of the XS4 genome are encoded using a similar genetic code to that of *Mycoplasma* (NCBI’s translation table 4). Therefore, DFAST was run with genetic code option 11 for RS3 and genetic code option 4 for XS4.

### Phylogenetic ana­lyses of 105 genes

We conducted two phylogenomic ana­lyses: the first including the broad member of gammaproteobacterial species as operational taxonomic units (OTUs) and the second focusing specifically on species of the families *Francisellaceae* and *Fastidiosibacteraceae* as well as their close relatives. In both ana­lyses, multiple sequence alignments were generated based on the set of 120 phylogenetically informative marker genes (bac120) provided in GTDB r214. In the first ana­lysis, we selected one representative genome from each order within *Gammaproteobacteria* and one representative genome from each genus within ‘*Francisellales*’, the order employed within the GTDB. As outgroups, we included one representative genome each from *Alphaproteobacteria*, *Zetaproteobacteria*, and *Magnetococcia*. In cases where a lineage lacked a representative genome or had multiple representative genomes, the genome with the highest completeness was selected. The bac120 homologs of RS3 and XS4 were manually selected from the results of BLASTp searches against the putative proteomes of RS3 and XS4, using the bac120 dataset of the other genomes as a query. Among the bac120 dataset, 105 proteins were detected in at least one of RS3 and XS4 and were used for downstream ana­lyses. The genomes and marker genes used in the ana­lysis are listed in Supplementary Table S2. We aligned each of the 105 protein sequences using MAFFT (version 7.490) with the L-INS-i method and trimmed ambiguously aligned positions with BMGE (version 2.0) using default settings (Criscuolo and Gribaldo, 2010). The remaining sites of the 105 proteins were concatenated into a single alignment comprising 203 taxa and 33,898 amino acid positions. We inferred the maximum likelihood (ML) tree based on the alignment by using IQ-TREE (version 2.2.2.5) (Minh *et al.*, 2020) under the LG+C10+F+I+G model, which was selected by ModelFinder of IQ-TREE (Kalyaanamoorthy *et al.*, 2017) with the “-m TEST -mset LG+C10” option. Statistical support for each bipartition in the ML tree was calculated by 1000-replicate ultrafast bootstrap approximations (Hoang *et al.*, 2018).

In the second phylogenomic ana­lysis, we selected one genome from each species within *Francisellaceae*, *Fastidiosibacteraceae*, ‘*Piscirickettsiales*’, and related environmental clades that were closely related to RS3 and XS4 in the first phylogenomic tree, totaling 44 genomes. The same 105 proteins used in the first ana­lysis were retrieved from the 44 genomes (Supplementary Table S3), as well as the RS3 and XS4 genomes, and the sequences were aligned and trimmed in the same manner as in the first ana­lysis. The 105 genes were concatenated into a single alignment comprising 46 taxa and 36,688 amino acid positions. The ML tree‍ ‍was inferred using IQ-TREE (version 2.2.6) under the LG+C60+F+I+G model, which was selected by ModelFinder with the “-m TEST -mset LG+C60” option. Statistical support for each bipartition in the ML tree was calculated by a 100-replicate non-parametric bootstrap ana­lysis with the ML tree used as the guide for estimating PMSF parameters (Wang *et al.*, 2018).

### Orthologous protein ana­lysis and functional annotation of proteins

Orthologous relationships between the proteins encoded in the RS3 and XS4 genomes and those of other *Fastidiosibacteraceae* bacteria were estimated using OrthoFinder (version 2.5.3) (Emms and Kelly, 2019). The OrthoFinder ana­lysis included all proteins from RS3 and XS4, as well as those from eight species belonging to five genera of *Fastidiosibacteraceae* bacteria (Supplementary Table S4). To infer the metabolic functions associated with each orthologous protein group (orthogroup), one representative protein sequence was extracted from each orthogroup, creating a single protein set comprising 2,935 sequences. This protein set was analyzed using the KEGG Automatic Annotation Server (KAAS) (Moriya *et al.*, 2007) to estimate the KEGG Orthology (KO) ID corresponding to each orthogroup (Kanehisa *et al.*, 2016). The search program used in the KAAS ana­lysis was BLAST, and the assignment method was the bi-directional best hit (BBH). The metabolic function of each orthogroup was inferred based on the KEGG annotations associated with the assigned KO IDs.

### Phylogenetic ana­lysis of the ADP:ATP antiporter

An orthologous protein ana­lysis revealed that the ADP:ATP antiporter found in RS3 and XS4 is not present in other closely related *Fastidiosibacteraceae* bacteria (Supplementary Table S5). To investigate the evolutionary origin of the ADP:ATP antiporter, we performed a phylogenetic ana­lysis. We searched for the amino acid sequences of the ADP:ATP antiporter in the NCBI refseq database as of July 9, 2024 by BLASTp searches using the ADP:ATP antiporter sequences of RS3/XS4 as queries. We retrieved subject sequences with E-values less than 1e-10, and divided the sequences into gammaproteobacterial, alphaproteobacterial, chlamydial, eukaryotic, and other sequences based on the lineages to which each sequence belongs. Redundancy within the subject sequences of each phylogenetic group was removed by individual clustering ana­lyses using CD-HIT (version 4.8.1) (Fu *et al.*, 2012) with a 70% identity threshold. We aligned the representative sequences of all lineages and ADP:ATP antiporter sequences of RS3/XS4 using MAFFT (version 7.525) with the L-INS-i method. Ambiguously aligned positions were trimmed by trimAl (version 1.4) (Capella-Gutiérrez *et al.*, 2009) with the “-gt 0.85” option. The final alignment comprised 229 sequences with 463 amino acid positions. We inferred the ML tree based on the alignment by using IQ-TREE (version 2.2.6) under the LG+C60+F+G model, which was selected by ModelFinder of IQ-TREE with the “-m TEST -mset LG+C60” option. Statistical support for each bipartition in the ML tree was calculated by 1000-replicate ultrafast bootstrap approximations.

## Results and Discussion

### Identification of circular genomes of RS3 and XS4

Nakayama *et al.* (2024) aimed to amplify the genome sequence of a cyanobacterium symbiotic with *C. regius* by obtaining whole-genome amplification products from a single *C. regius* cell (Nakayama *et al.*, 2024). Since the entire cell served as the template, the genomes of any organisms associated with this cell were theoretically amplified. Nakayama *et al.* (2024) focused on symbiotic cyanobacteria and extracted only the cyanobacterial genome. In the present study, we investigated whether the amplification product contained any microbes other than cyanobacteria. The results obtained revealed two circular chromosomes showing homology to *Gammaproteobacteria* from an assembly of the amplified genome using a single *C. regius* cell. The two gammaproteobacterial chromosomes have similar, but clearly distinguishable, rRNA gene sequences with 89.95% identity, suggesting that these chromosomes represent the genomes of two phylogenetically related, but separate bacteria (hereinafter referred to as RS3 and XS4). The genome of RS3 was 529‍ ‍kbp in size with a GC content of 33%, whereas the genome of XS4 was 436‍ ‍kbp in size with a GC content of 28%.

In addition to these bacterial genomes, we identified a potential complete archaeal genome sequence within the same assembly. This archaeal genome is exceptionally small and displays higher sequence divergence than any known archaeal genomes. We considered this genome to have originated from an archaeon representing a novel lineage. Therefore, discussing this archaeal genome alongside RS3 and XS4 within the same biological and evolutionary context is challenging and will be addressed in a separate publication.

### Genetic code in XS4

The preliminary annotation of the XS4 genome revealed locations where open reading frames were interrupted by UGA codons, which function as termination signals in the standard genetic code. Further ana­lyses revealed that these UGA codons corresponded to sites at which tryptophan residues are typically conserved in other bacteria and, thus, are likely translated as tryptophan in XS4 (Supplementary Fig. S1, see Materials and Methods). This ‘UGA=W’ reassignment has also been observed in bacteria with a low GC content in their genomes, including *Mycoplasma* and some arthropod endosymbionts (Knight *et al.*, 2001; McCutcheon and Moran, 2011). In the standard code, tryptophan is assigned by UGG, and the reassignment of UGA from translation termination to tryptophan may be attributed to selection pressure for a low GC content in the genome (Osawa *et al.*, 1992; Knight *et al.*, 2001). Therefore, the XS4 genome was annotated using the *Mycoplasma* genetic code (NCBI’s translation table 4).

### Genomic features and phylogeny of RS3 and XS4

**Fig. 1.**
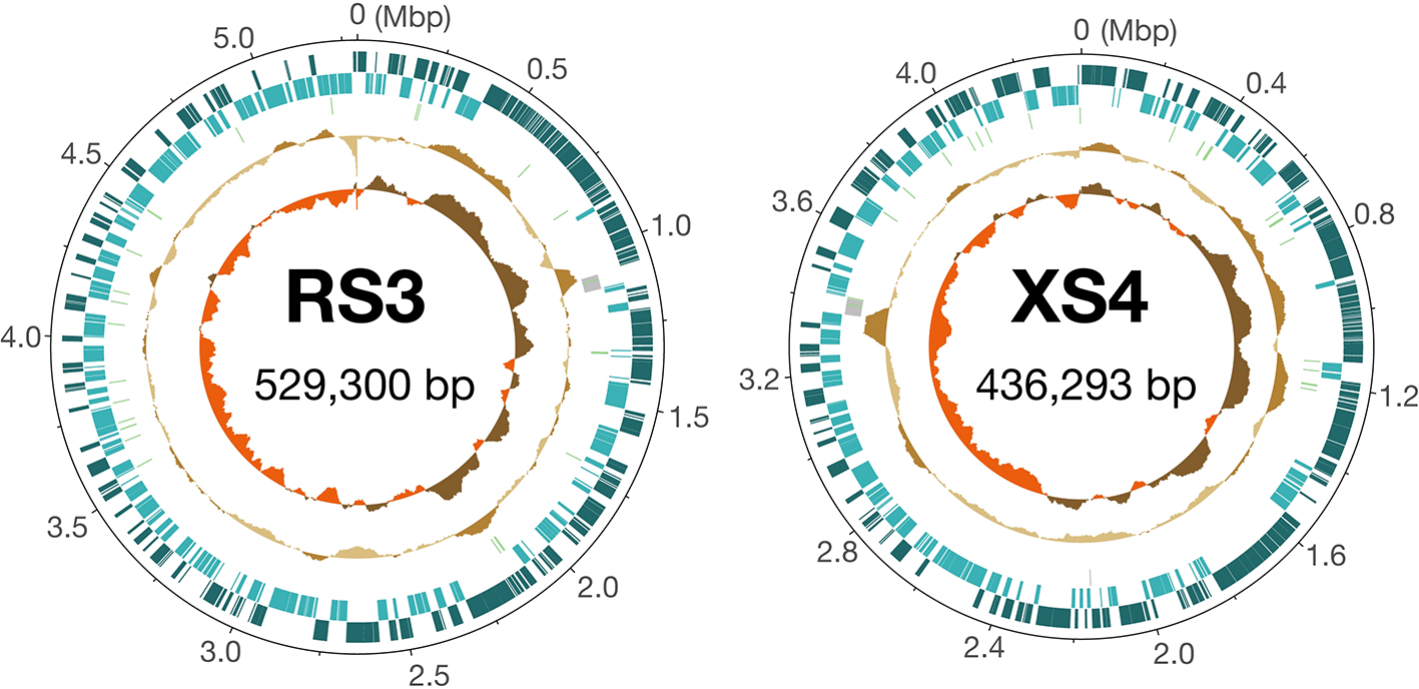
Genome maps of RS3 and XS4. In each map, the outer two circles indicate the positions of protein-coding genes on the plus and minus strands. The third circle shows the positions of the tRNA (green bars) and rRNA (grey bars) genes. The second innermost circle shows the relative G+C content and the innermost circle plots the GC skew.

The genome annotation of the two genomes revealed 495 protein-coding genes in the RS3 genome and 426 in the XS4 genome. Each genome contained one copy of the 16S, 23S, and 5S rRNA genes, as well as 32 tRNA genes on RS3 and 31 tRNA genes on XS4, along with one transfer-messenger RNA (tmRNA) gene on both genomes (Fig. 1). While the possibility of additional genomic elements, such as plasmids, cannot be excluded, the detection of rRNA genes and a complete set of tRNA genes corresponding to all 20 amino acids on both circular chromosomes strongly suggests that the genome sequences identified in this study represent the complete genomes of RS3 and XS4. Since typical bacterial genomes are several million base pairs in size, the genomes of RS3 and XS4, which are 529 and 436‍ ‍kbp, respectively, are very compact.

Previous studies detected *nifH* genes and 16S rRNA genes from bacteria other than cyanobacteria in *Dinophysales* dinoflagellates, including *Citharistes* sp. (Foster *et al.*, 2006; Farnelid *et al.*, 2010). The RS3 and XS4 genomes revealed in the present study lack nitrogen fixation-related genes, including *nifH*. Therefore, RS3 and XS4 may not have been detected in the survey of bacteria associated with *Dinophysales* cells using *nifH* sequences (Farnelid *et al.*, 2010). Comparisons with the 16S rRNA gene sequences obtained from *Dinophysales* cells in a previous study (Foster *et al.*, 2006) were not possible because the non-cyanobacterial sequences obtained were not deposited in public databases.

**Fig. 2.**
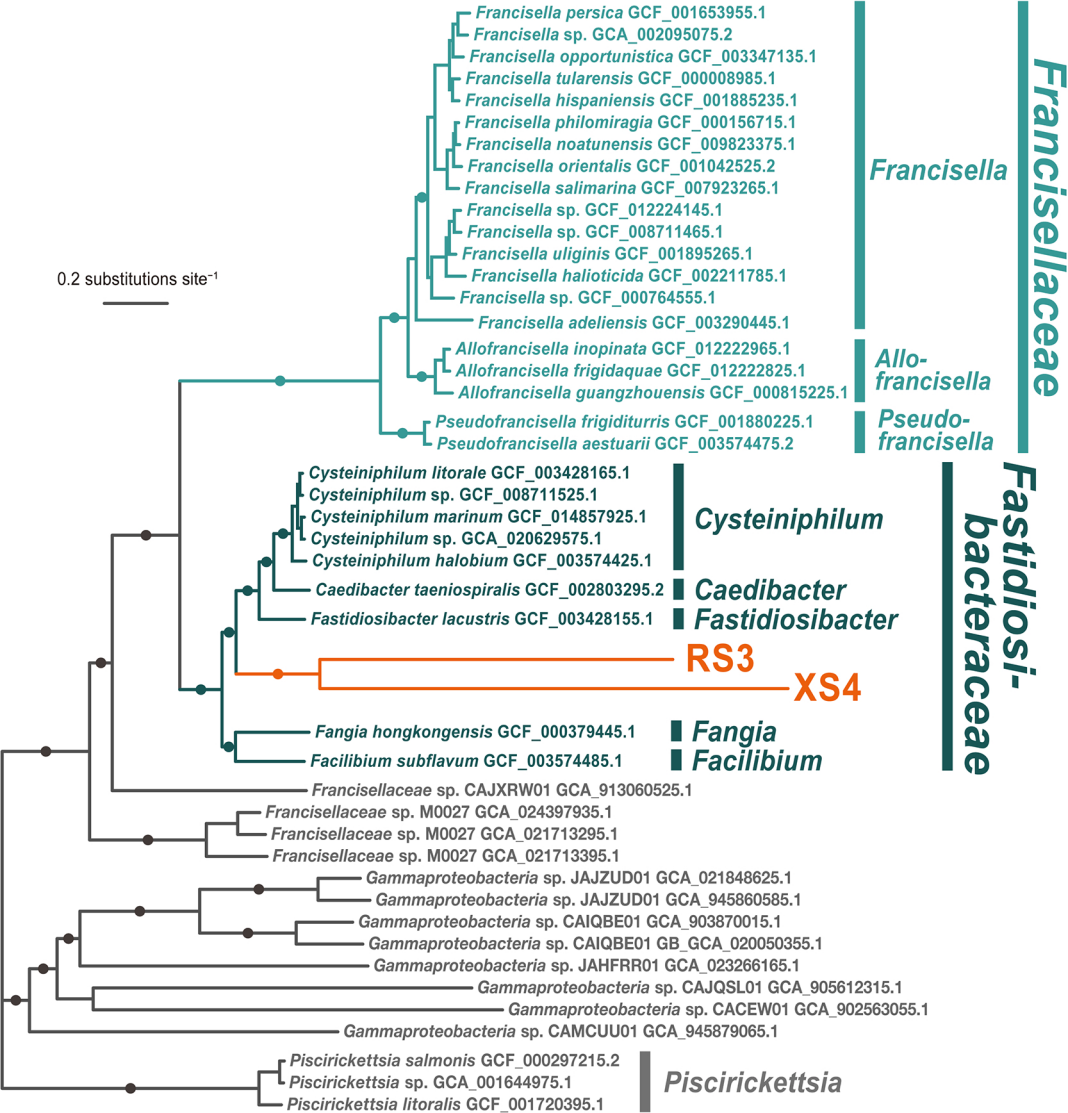
Maximum likelihood phylogenetic tree based on 105 protein sequences. The tree was inferred using IQ-Tree under the LG+C60+F+I+G model. Non-parametric bootstrap values (100 replicates) were mapped on the tree. The nodes marked with filled circles were supported with 100% bootstrap values. The alignment included 46 taxa and 36,688 amino acid positions.

A multigene phylogenetic ana­lysis was conducted to infer the phylogenetic positions of RS3 and XS4 among bacterial diversity. The preliminary sequence similarity search suggested that both of these bacteria are related to species in the families *Fastidiosibacteraceae* and *Francisellaceae* of *Gammaproteobacteria*. We reconstructed a phylogenetic tree using genome sequences covering the diversity of *Gammaproteobacteria*, including *Fastidiosibacteraceae* and *Francisellaceae*, based on 105 protein sequences, which are highly conserved across bacterial lineages (Supplementary Fig. S2). The topology of the tree suggested that RS3 and‍ ‍XS4 are both closely related to species within *Fastidiosibacteraceae*. To gain further insights into the precise phylogenetic positions of RS3 and XS4, an additional phylogenetic ana­lysis was conducted using a dataset including a more comprehensive sampling of fastidiosibacteracean and francisellacean species. Fig. 2 shows the resulting ML phylogenetic tree, revealing that RS3 and XS4 constitute a monophyletic group within a cluster comprising *Fastidiosibacteraceae* bacteria. The lineage of RS3 and XS4 was shown to be a sister clade to the monophyletic group formed by the genera *Cysteiniphilum*, *Caedibacter*, and *Fastidiosibacter*. This monophyletic group, along with the clade comprising the genera *Fangia* and *Facilibium*, represents the monophyletic lineage of fastidiosibacteraceans. Additionally, a clade of *Francisellaceae* bacteria was inferred as a sister clade to the *Fastidiosibacteraceae* lineage. All the aforementioned phylogenetic relationships were robustly supported (bootstrap values of 100%).

The branches leading to RS3 and XS4 from the node connecting them were longer than those of other fastidiosibacteraceans, indicating a rapid evolutionary rate for these bacterial genomes. This topology of the tree is reminiscent of the Long Branch Attraction (LBA) artifact, which is a methodological artifact where rapidly evolving lineages are incorrectly inferred to be closely related. However, since the present ana­lysis employed a mixture model accounting for the site heterogeneity of amino acids frequencies, which is more robust to LBA (Quang *et al.*, 2008), we considered this tree topology to represent the most plausible evolutionary hypothesis to date. The topology suggests that RS3 and XS4 are the closest relatives, diverging from a common ancestor shared with the genera *Cysteiniphilum*, *Caedibacter*, and *Fastidiosibacter*.

In the context of symbiotic relationships, including parasitism, *Caedibacter*, a member of the *Fastidiosibacteraceae* clade along with RS3 and XS4, is noteworthy. The representative species *Caedibacter taeniospiralis* is an obligate cytoplasmic endosymbiont found in *Paramecium*, known for imparting toxic traits, referred to as “killer traits”, to infected *Paramecium* against non-infected cells (Kusch *et al.*, 2000; Grosser *et al.*, 2018). Although most members of *Fastidiosibacteraceae* have been discovered in the past two decades, the research on *Paramecium* killer traits dates back to the mid-20th century (Sonneborn, 1943; Preer *et al.*, 1974), making *Caedibacter* the most well-known genus within this clade. The dinoflagellate *C. regius*, the source of the genome amplification product from which the RS3 and XS4 genomes were identified, is a protist belonging to Alveolata, as is *Paramecium*, implying a similar symbiotic association. Nevertheless, our phylogenetic ana­lysis revealed that the *Caedibacter* and RS3/XS4 lineages were not closely related. In addition to *Caedibacter*, the marine fastidiosibacteracean genus *Cysteiniphilum*, which has been reported to parasitize humans (Xu *et al.*, 2021), is not the closest relative of the RS3/XS4 lineage, but is a sister clade to *Caedibacter*.

### Genome reduction and codon reassignment

**Fig. 3.**
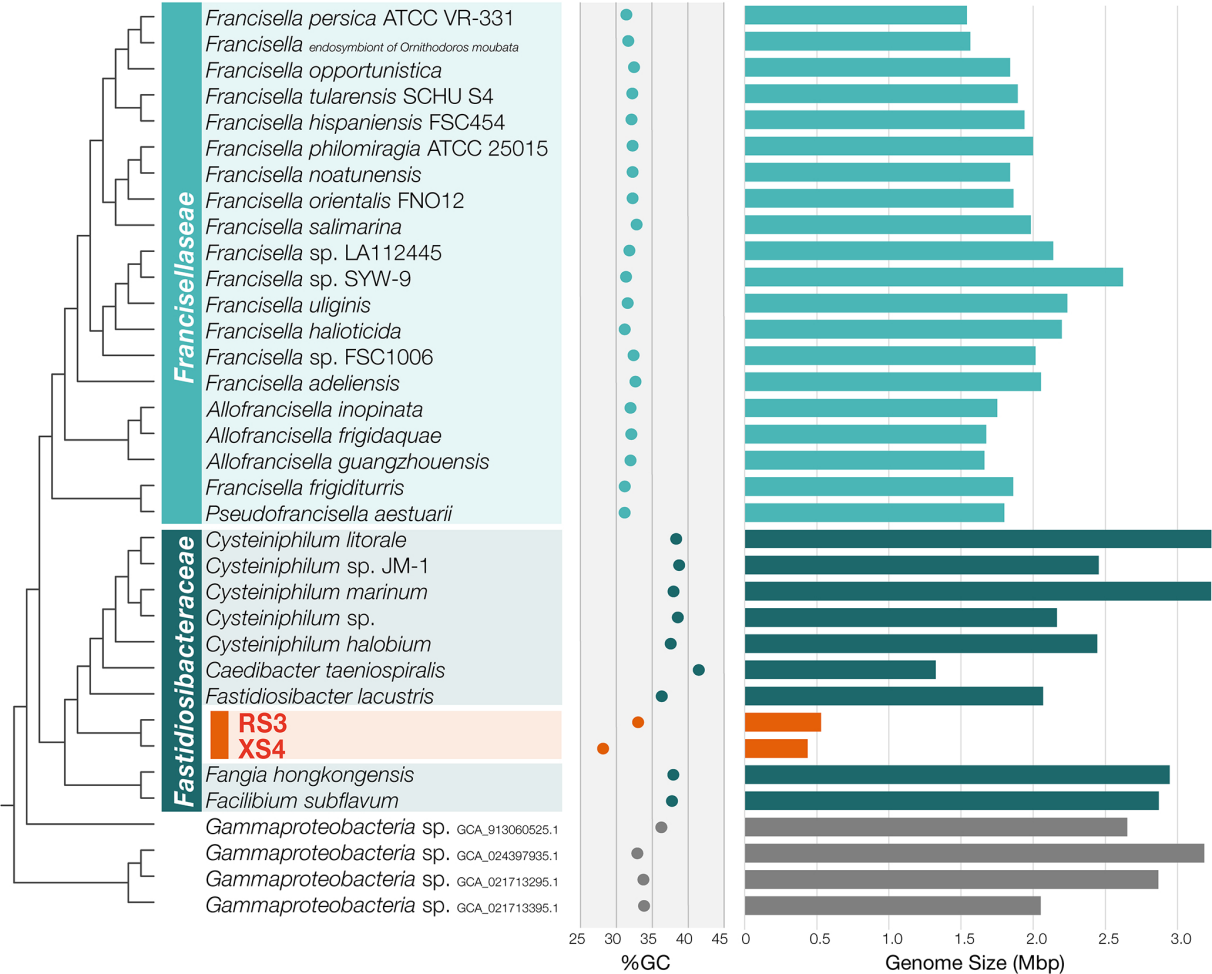
Comparison of the size and GC content of genomes of RS3, XS4, and their related bacteria. The cladogram on the left side shows phylogenetic relationships as shown in Fig. 2. The bar graph on the right displays genome sizes, while the plot in the center indicates the GC content of each genome.

A comparative ana­lysis of genome sizes based on the evolutionary relationships inferred by the phylogenomic tree revealed marked reductions in the genome sizes of RS3 and XS4. Fig. 3 shows a comparison of the genome size and GC content of each bacterial genome along with phylogenetic relationships; the genome sizes of *Fangia* and *Facilibium*, which represent the most basal branches of the *Fastidiosibacteraceae* lineage, are both slightly less than 3‍ ‍Mbp, while those of *Cysteiniphilum* and *Fastidiosibacter*, which are members of the sister clade of the RS3/XS4 lineage, range from 2–3‍ ‍Mbp. In consideration of the genome sizes of the bacteria surrounding RS3 and XS4 in the phylogeny, the ancestral bacteria of RS3 and XS4 appeared to possess a genome of at least 2‍ ‍Mbp. In contrast, the genome sizes of RS3 and XS4 are 529 and 436‍ ‍kbp, respectively, indicating that their genomes have undergone severe reductions to less than 25% of their ancestral size. Although the obligate cytoplasmic endosymbiont *C. taeniospiralis* is also known to possess a relatively compact genome, it is more than twice the size of the RS3/XS4 genomes.

Another notable feature shared by RS3 and XS4 is their low GC content (Fig. 3). The GC content of the RS3 genome of 33.04% is lower than those of other bacterial genomes of *Fastidiosibacteraceae*, except for XS4. The XS4 genome exhibits an even lower GC content of 28.16%, which is less than those observed in the genomes of *Francisellaceae* bacteria or those of fastidiosibacteracean genomes. Previous genomic studies demonstrated a universal bias towards a lower GC content in reduced genomes (McCutcheon and Moran, 2011), and a similar evolutionary change appears to have occurred in the genomes of RS3 and XS4. Changes in the GC content in reduced genomes are consistent with the result showing that the XS4 genome, with its lower GC content, was smaller than the RS3 genome.

In bacterial genomes that underwent severe size reductions and received a strong bias towards a low GC content, UGA has often been reassigned from the termination signal to tryptophan (Ohama *et al.*, 2008). XS4 most likely uses the deviant genetic code, the “UGA=W code”, which assigns UGA to tryptophan, while RS3 uses the standard code (*i.e.*, UGA as a termination signal). In bacteria with the standard code, two class-I release factors (*i.e.*, RF1 and RF2), which promote the release of the nascent peptide from the ribosome responding to a stop codon at the A site, bear distinct codon specificities: RF1 recognized UAA and UAG, and RF2 recognized UAA and UGA (Caskey *et al.*, 1969). Therefore, there are two possibilities for RF2 in XS4. XS4 may have discarded RF2 during the UGA reassignment because RF1 is sufficient to cover UAA and UAG in this bacterium. Alternatively, RF2 persists, but may have lost codon specificity to UGA in XS4. The XS4 genome possesses the gene encoding RF2 and, thus, this protein is anticipated to be specific for UAA. We compared the putative amino acid sequence of RF2 in XS4 to that of RF2 in RS3 (note that the latter is assumed to recognize both UAA and UGA as termination signals). Unfortunately, the comparison provided no insights into the amino acid(s) that caused the change in codon specificity in RF2 in XS4 (Supplementary Fig. S3). The amino acids of RF2, which were shown to interact directly with the UGA and UAA triplets in the crystal of the translation termination complex comprising the ribosome, a triplet (UAA or UGA), and RF2 (Korostelev *et al.*, 2008; Weixlbaumer *et al.*, 2008), were conserved in both XS4 and RS3 proteins.

**Fig. 4.**
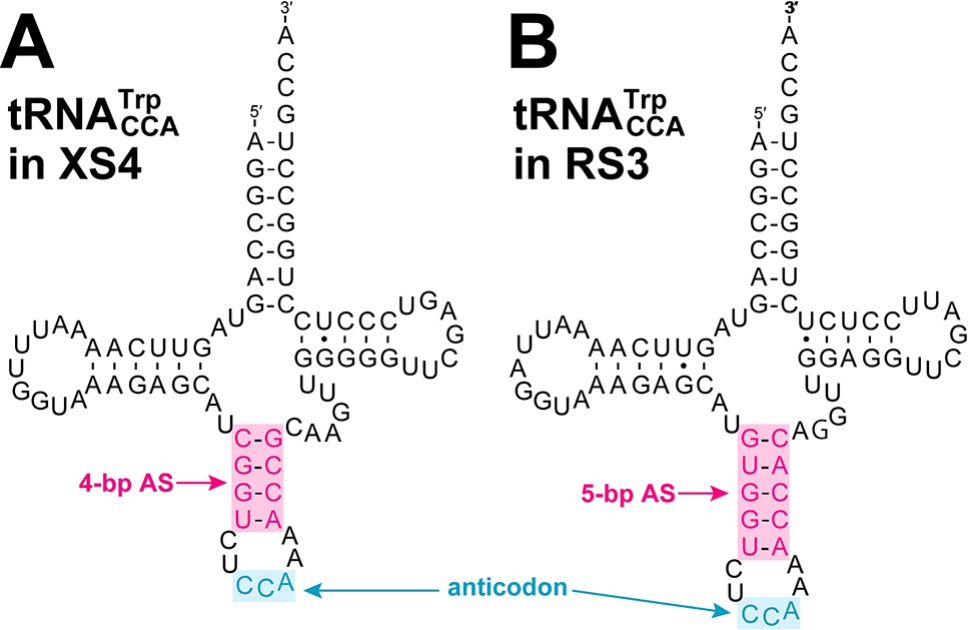
Predicted secondary structures of tRNA${}_{\mathrm{CCA}}^{\mathrm{Trp}}$. The anticodon stem of tRNA in the XS4 genome (A) comprised four base pairs (4-bp AS), while that of the RS3 genome (B) was composed of five base pairs (5-bp AS).

Responding to the UGA reassignment, XS4 requires a tryptophan tRNA (tRNA^Trp^), of which the anticodon binds to UGA. We found only a single tRNA^Trp^ with the anticodon CCA (tRNA${}_{\mathrm{CCA}}^{\mathrm{Trp}}$) in both the XS4 and RS3 genomes. While the CCA anticodon theoretically cannot translate UGA because cytosine in the first anticodon position forms only a Watson-Crick pair with guanine in the third codon position, experiments on *Escherichia coli* tRNA${}_{\mathrm{CCA}}^{\mathrm{Trp}}$ demonstrated that this tRNA translates UGA codons with low efficiency (Hirsh, 1971; Weiner and Weber, 1971). A recent study on the parasitic protist, *Blastocrithidia nonstop*, in which UGA is used as a tryptophan codon, demonstrated that the length of the anticodon stem of tRNA${}_{\mathrm{CCA}}^{\mathrm{Trp}}$ was critical for the translation of UGA (Kachale *et al.*, 2023). In eukaryotes with the standard code (*e.g.*, *Saccharomyces cerevisiae*), the anticodon stem of tRNA${}_{\mathrm{CCA}}^{\mathrm{Trp}}$ comprises five base pairs (5-bp AS) and binds solely to UGG. In contrast, *B. nonstop* has tRNA‍${}_{\mathrm{CCA}}^{\mathrm{Trp}}$ with the anticodon stem composed of four base pairs (4-bp AS) and the efficient translation of UGA by this non-canonical tRNA${}_{\mathrm{CCA}}^{\mathrm{Trp}}$ has been demonstrated experimentally (Kachale *et al.*, 2023). The length of the anticodon stem in tRNA${}_{\mathrm{CCA}}^{\mathrm{Trp}}$ and UGA assignments between XS4 and RS3 are consistent with this finding. We found tRNA${}_{\mathrm{CCA}}^{\mathrm{Trp}}$ with a 4-bp AS in XS4 with the “UGA=W code”, while the same tRNA with a 5-bp AS was detected in RS3 with the standard code (Fig. 4). Collectively, these results suggest that tRNA${}_{\mathrm{CCA}}^{\mathrm{Trp}}$ with a 4-bp AS recognizes UGA as a tryptophan codon, in addition to UGG, in XS4.

### Reductive evolution of metabolic capacities

**Fig. 5.**
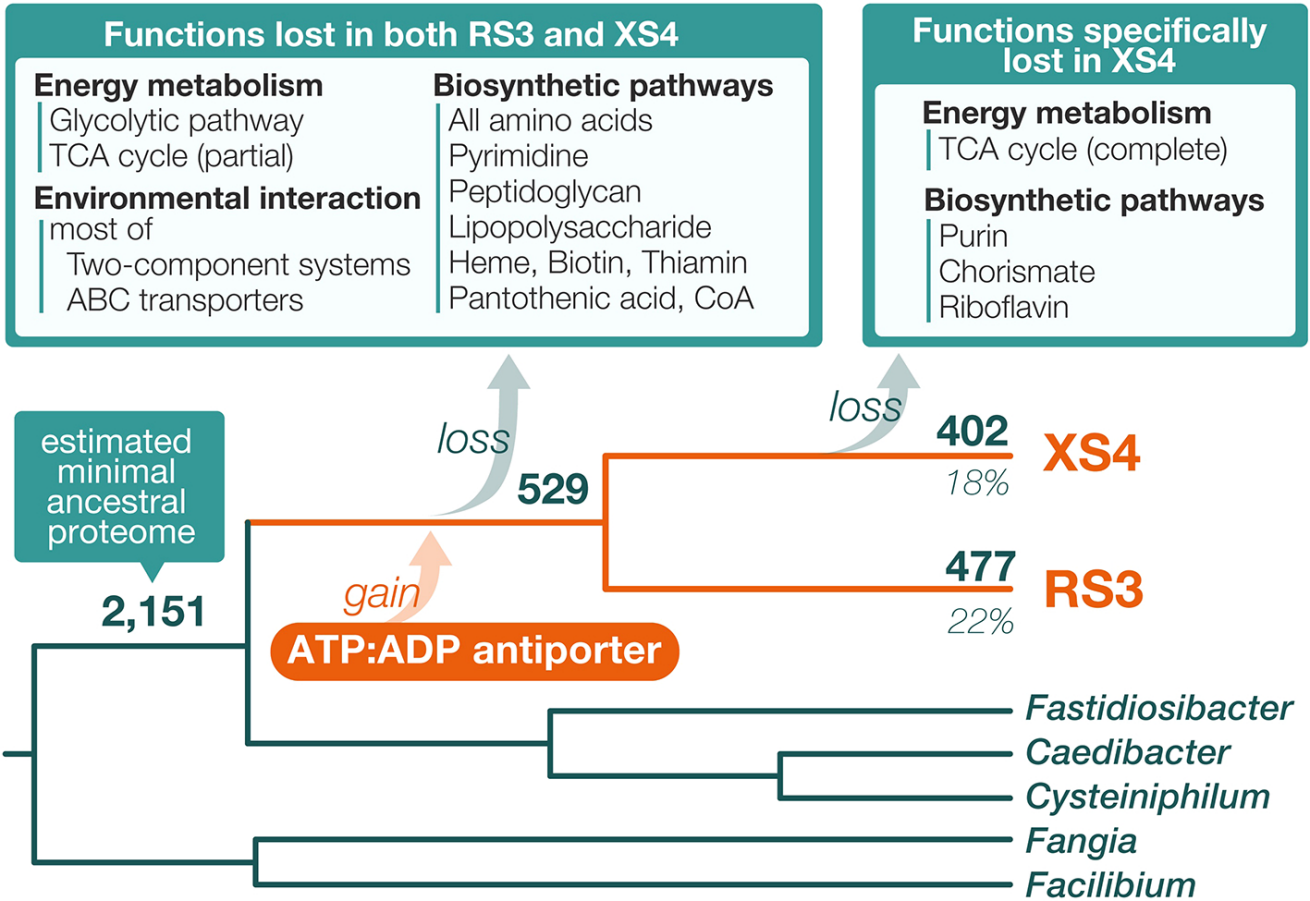
Predicted evolution of metabolic functions in the lineage of RS3 and XS4. Numbers above branches indicate the number of proteins derived from the minimal ancestral proteome (2,151 proteins), which was estimated based on the orthologous protein ana­lysis. The percentages below the tips of the branches for RS3 and XS4 represent the percentage of proteins retained from the minimal ancestral proteome in each genome. Metabolic functions lost or gained during the evolutionary process of each bacterium are shown.

Reflecting their small genome sizes, the genomes of RS3 and XS4 encode a correspondingly limited number of proteins: 495 and 426 protein-coding genes, respectively. In contrast, other *Fastidiosibacteraceae* bacteria, including species of *Cysteiniphilum*, *Facilibium*, *Fangia*, and *Fastidiosibacter*, have approximately 2,000–3,000 protein-coding genes in their genomes. Even the relatively compact genome of *C. taeniospiralis* contains 1,218 proteins. We conducted a comprehensive ana­lysis of orthologous relationships between the proteins of RS3/XS4 and those of related bacteria to infer the evolution of the RS3 and XS4 proteomes in the light of the organismal phylogenetic relationship. An orthologous protein ana­lysis revealed that nearly all proteins observed in the RS3/XS4 genomes (RS3: 480 out of 495 proteins, XS4: 408 out of 426 proteins) possess orthologous counterparts in their closely related bacterial species. By examining the proteomes of the phylogenetically related taxa, we inferred the minimal proteome of the common ancestor of RS3 and XS4. This ancestral proteome was defined as the set of proteins that satisfies two‍ ‍criteria: the proteins found in 1) either *Fangia* or *Facilibium*, representing the most basal branch of the *Fastidiosibacteraceae* clade, and 2) at least one member of the sister lineage to RS3/XS4, comprising *Cysteiniphilum*, *Fastidiosibacter*, or *Caedibacter*. The minimal ancestral proteome was estimated to comprise 2,151 proteins, with RS3 and XS4 retaining 477 and 402 of these proteins, respectively, which corresponds to only 22.1 and 18.6%, respectively, of the ancestral proteome. Additionally, we assigned functional annotations using the KO database (Kanehisa *et al.*, 2023) to each orthologous protein group in order to infer the metabolic capacities of RS3 and XS4 and the evolution of their proteomes. In comparisons with the ancestral proteome, the majority of the metabolic functions lost were shared between RS3 and XS4 (Supplementary Table S5), suggesting that these losses occurred at the stage of their common ancestor (Fig. 5). Among the lost metabolic functions, transporters and two-component regulatory systems were the most affected functional categories, which are typically lost in symbiotic bacteria (Shigenobu *et al.*, 2000; McCutcheon and Moran, 2011). RS3 and XS4 both lacked biosynthetic pathways for essential metabolites that are indispensable for independent growth. This was particularly evident in the biosynthesis of amino acids, where both bacteria lost all major amino acid biosynthesis pathways. Additionally, both bacteria lacked genes for the synthesis of pyrimidine bases, heme, the majority of vitamins, most glycolytic enzymes, and 50% of TCA cycle enzyme genes. Moreover, XS4, with a more reductive genome than RS3, lost additional metabolic pathways, including biosynthetic pathways for purine bases, chorismic acid, and riboflavin, and the entire set of the remaining TCA cycle enzyme genes, which were conserved in RS3. This extensive loss of metabolic functions indicates that RS3 and XS4 rely on their host for a significant amount of the metabolites required for their growth. Moreover, a distinguishing feature of RS3 and XS4 is the absence of biosynthetic pathways for peptidoglycan and lipopolysaccharide (LPS). While the morphologies and symbiotic natures of these bacteria remain unclear, the loss of these key cell surface components suggests that both bacteria are intracellular symbionts (or parasites).

A number of examples in which intracellular symbiotic bacteria synthesize and supply metabolic products that the host organism is unable to produce have been reported (McCutcheon and Moran, 2011). In such cases, the host and symbiont both benefit from the relationship. Nevertheless, the relationship between RS3/XS4 and their host organisms is not necessarily analogous to the aforementioned cases. A lack of knowledge of the metabolic characteristics of the presumed host, *C. regius*, makes it challenging to discuss the relationship in detail. However, RS3/XS4 lost almost all pathways for synthesizing amino acids and vitamins, which makes it unlikely that they produce and supply the necessary substances for the host. On the other hand, it cannot be entirely ruled out that the residual biosynthetic pathways in RS3/XS4, such as those for folic acid and fatty acids, still confer benefits to the host. This possibility may be exami­ned once the metabolic functions of *C. regius* are elucidated in future studies. Regardless, it is reasonable to assume that RS3/XS4 rely heavily on their host for the majority of the substances constituting their cells, characterizing these bacteria as primarily nutritional parasites. In this context, a protein-coding gene of interest has been identified in the genomes of these two bacteria. The ADP:ATP antiporter (RS3CREG01_2930 and XS4CREG01_3620; KO ID: K03301), which was detected from the proteomes of RS3 and XS4, was not observed in any proteomes of *Fastidiosibacteraceae* bacteria exami­ned in the present study and, thus, was not included in the estimated ancestral proteome of RS3/XS4. A phylogenetic ana­lysis of the antiporter from various bacteria as well as those of RS3 and XS4 (Supplementary Fig. S4) strongly supported the monophyletic relationship of the proteins from RS3 and XS4. Additionally, the RS3/XS4 clade was shown to be monophyletic with alphaproteobacterial sequences; however, the relationship was weakly supported. While homologous proteins were also identified from gammaproteobacterial species other than fastidiosibacteraceans and francisellaceans, none of these were suggested to be closely related to the RS3/XS4 sequences (Supplementary Fig. S4). The phylogenetic relationship indicates that the common ancestor of RS3/XS4 acquired the ADP:ATP antiporter gene through horizontal gene transfer; however, the donor has yet to be identified (Fig. 5). It is important to note that several notorious intracellular parasitic bacteria, such as *Chlamydia* and *Rickettsia*, are known to engage in energy parasitism. These parasites utilize the ADP:ATP antiporter as a key molecule for obtaining energy from their host organisms (Schmitz-Esser *et al.*, 2004). Given these characteristics, the acquisition of this gene may enable RS3/XS4 to engage in energy parasitism. Similar genomic features, such as marked genome reductions and the presence of horizontally acquired ADP:ATP antiporters, have been reported in the denitrifying endosymbiont of the anaerobic ciliate, *Candidatus* Azoamicus ciliaticola (Graf *et al.*, 2021), and related bacteria capable of both denitrification and aerobic respiration (Speth *et al.*, 2024). However, in contrast to RS3/XS4, these bacteria are considered to function primarily as respiratory symbionts, generating ATP via their respiratory chains and providing it to their hosts. Although RS3/XS4 also encode genes for respiration (*e.g.*, genes for NADH dehydrogenase and ATP synthase), we did not detect the metabolic pathways typically involved in generating sufficient reducing equivalents for the electron transport chain, including the denitrification pathway. This suggests that RS3/XS4 followed a different evolutionary trajectory from these respiratory symbionts.

## Conclusion

We herein report the genomes of RS3 and XS4, which were identified using amplified DNA obtained from the dinoflagellate *C. regius* cell. RS3 and XS4 are regarded as novel symbiotic lineages belonging to *Fastidiosibacteraceae*, *Gammaproteobacteria*. These bacteria possess highly reduced genomes (529‍ ‍kbp for RS3 and 436‍ ‍kbp for XS4), which, to the best of our knowledge, are the smallest among the genomes sequenced at the chromosomal level within both *Fastidiosibacteraceae* and the encompassing order *Beggiatoales* (or *Thiotrichales*). Predicted proteomes of the two bacteria strongly suggest that RS3 and XS4 are adapted to an intracellular lifestyle. Furthermore, their markedly constrained biosynthetic capabilities indicate that these bacteria exist primarily as nutritional parasites within host cells. However, further research is needed to confirm their physical relationship with *C. regius*. Specifically, fluorescence *in situ* hybridization (FISH) or other microscopic techniques are required to visualize the relationship of RS3/XS4 with the *C. regius* cell. The genomic characteristics of RS3 and XS4 revealed in this ana­lysis not only expand our under­standing of genome diversity within *Fastidiosibacteraceae*, but also contribute to our knowledge of the phylogenetic and genomic diversities of symbiotic bacteria in protists. Further studies on bacteria symbiotic with protists are anticipated to yield even more insights.

## Citation

Nakayama, T., Harada, R., Yabuki, A., Nomura, M., Shiba, K., Inaba, K., and Inagaki, Y. (2025) Marked Genome Reduction Driven by a Parasitic Lifestyle: Two Complete Genomes of Endosymbiotic Bacteria Possibly Hosted by a Dinoflagellate. *Microbes Environ ***40**: ME25005.

https://doi.org/10.1264/jsme2.ME25005

## Supplementary Material

Supplementary Material 1

Supplementary Material 2

Supplementary Material 3

Supplementary Material 4

Supplementary Material 5

Supplementary Material 6
