# Supplementary material for: Marked Genome Reduction Driven by a Parasitic Lifestyle: Two Complete Genomes of Endosymbiotic Bacteria Possibly Hosted by a Dinoflagellate
Source: Microbes Environ. 2025 Jun 5;40(2):ME25005. doi: 10.1264/jsme2.ME25005 (PMC12213064; doi:10.1264/jsme2.ME25005)
Supplement: Supplementary file 1 — Supplementary Material 1 [file 40_25005_s1.pdf]

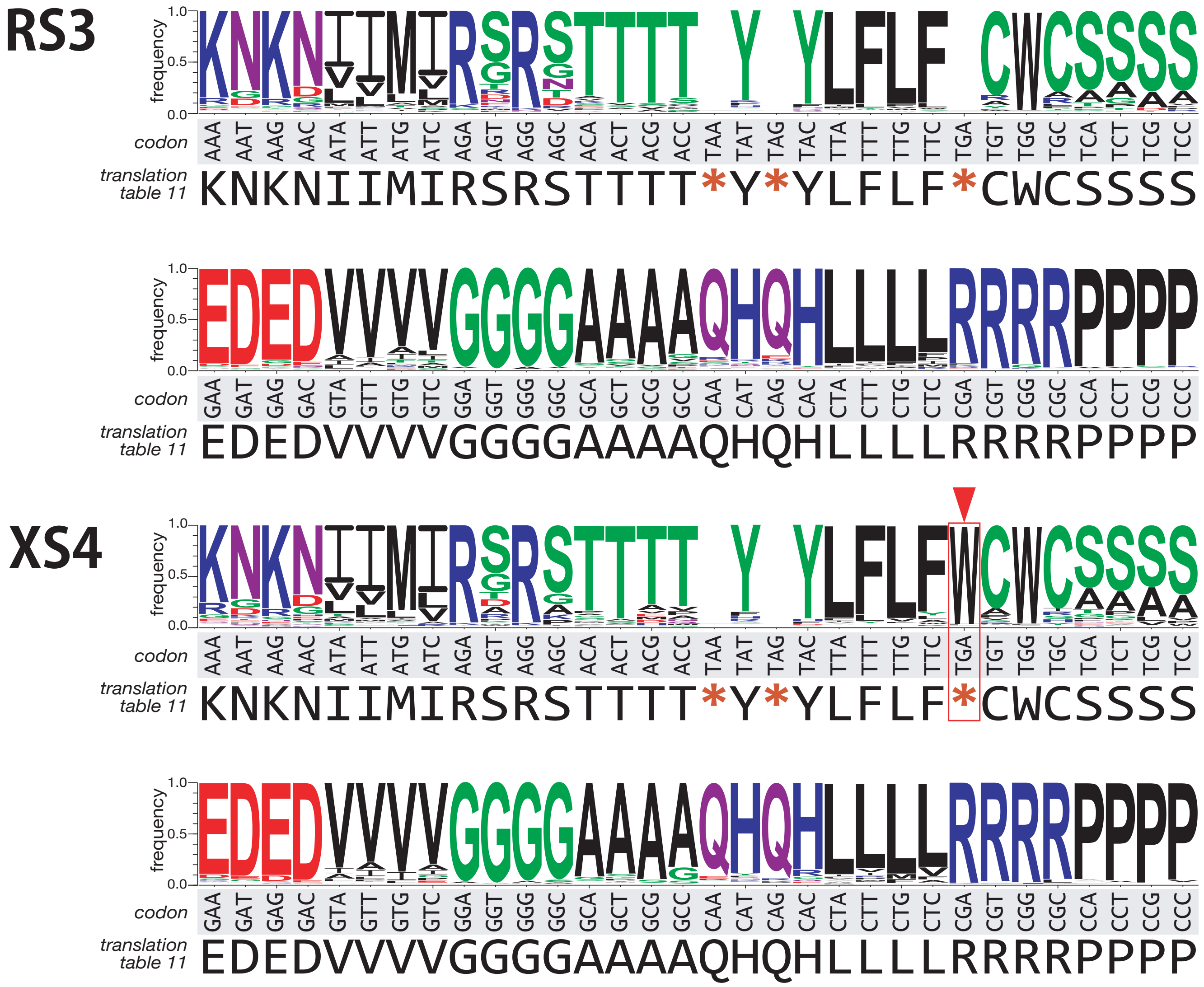

Supplementary figure S1. Relationship between codons in the RS3 and XS4 genomes and corresponding orthologous amino acids in other bacteria. The logoplot shows the proportion of amino acids observed at orthologous protein sites in bacterial sequences for each codon (DNA triplets) of putative protein-coding regions in the RS3 and XS4 genomes. The analysis focused on highly conserved sites where at least 90% of bacterial sequences, excluding RS3 and XS4, exhibited the same amino acid. The most frequently observed amino acid for each codon matched translations based on NCBI's translation table 11. Similarly, in XS4, the results were largely consistent, although the amino acid site corresponding to TGA (UGA in mRNA) in other organisms predominantly exhibited tryptophan (W), suggesting that in XS4, TGA (UGA) codes for tryptophan instead of serving as a stop codon.

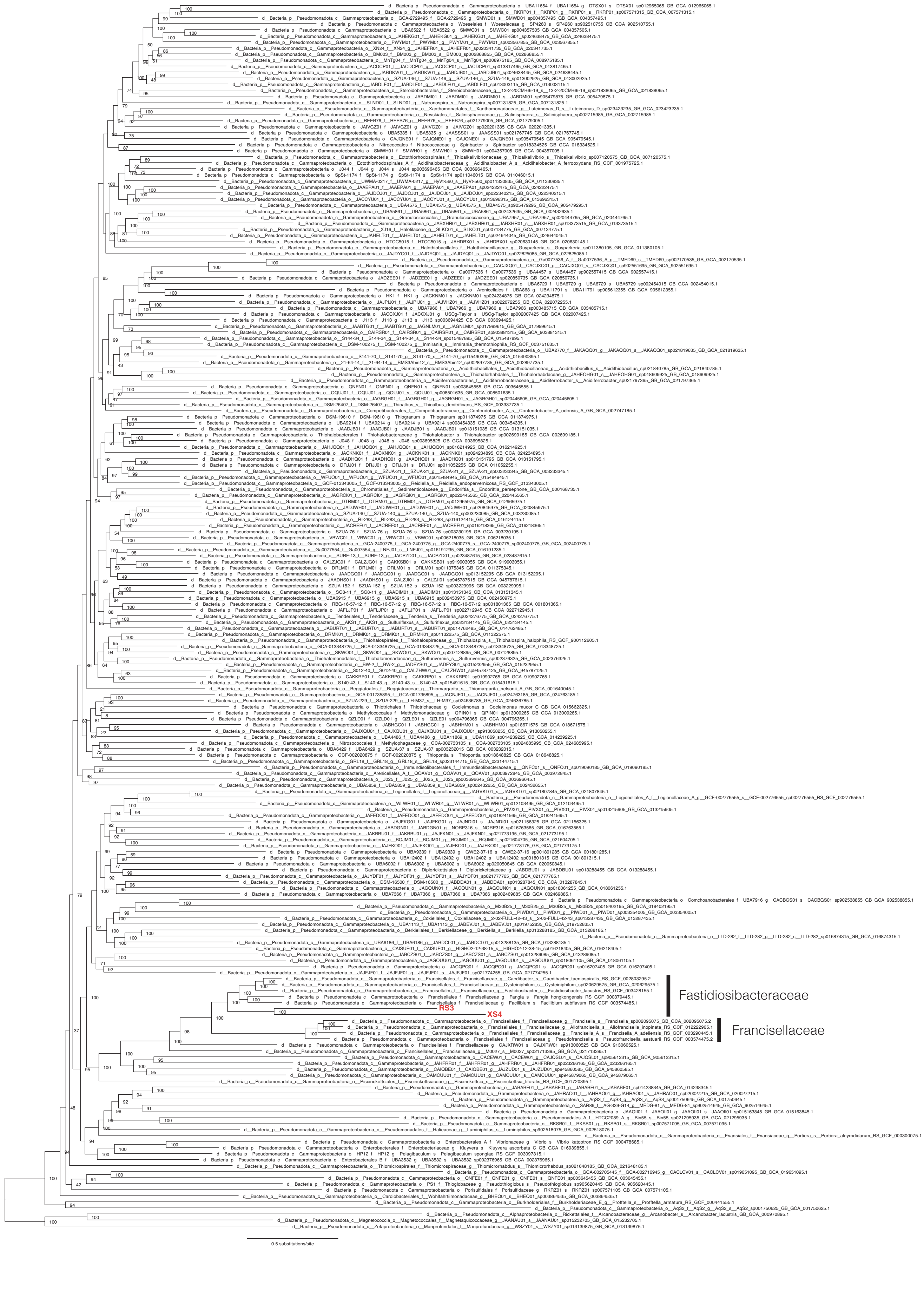

Supplementary figure S2. Maximum likelihood phylogenetic tree based on 105 protein sequences with broad taxon sampling. The tree was inferred using IQ-Tree under the LG+C10+F+I+G model. The statistical support for each bipartition in the ML tree was calculated by 1000-replicate ultrafast bootstrap approximation. The alignment included 203 taxa and 33,898 amino acid positions. The labeling of leaves is in accordance with the GTDB classification system.

```
XS4 RF2      ------------------------------------------------------------
RS3 RF2      MKNEKNNKEFILQTKNNAYKSKLIDMKRRIIVIENISKIKTKEKRLEEIFKELESPKIWE
E.coli RF2   M------------FEINPVNNRIQDLTERSDVLRGYLDYDAKKERLEEVNAELEQPDVWN

XS4 RF2      ---------------------MNSLIDAVGTVSDLVSFSAQEKDDSIIEDILLEINNIKI
RS3 RF2      NKDYAKNINQEKINLQKTINNIKTLKNEVHEKLELLNFATENYEDFFFAEIIQEIHIFEK
E.coli RF2   EPERAQALGKERSSLEAVVDTLDQMKQGLEDVSGLLELAVEADDEETFNEAVAELDALEE

XS4 RF2      QLKRLELTCLFSNPLDSNNAFLDIQSGSGGTEAQDWAQILMRMYIRWGESHSFRVAITDI
RS3 RF2      KLKKFELVRKFSRNIDFNNAFLDIQSGSGGIEAQDWAQMLMRMYLKWGESHGFKTEITNI
E.coli RF2   KLAQLEFRRMFSGEYDSADCYLDIQAGSGGTEAQDWASMLERMYLRWAESRGFKTEIIEE

XS4 RF2      SHGEIAGIKSCTIHFMGKYSYGLLRTETGIHRLVRKSPFDSGNRRHTSFASVLSFPEIKD
RS3 RF2      KEGDVAGIKNCTISFKGEYAFCLLRTETGVHRLVRKSPFDASNRRHTSFASIFISPEINK
E.coli RF2   SEGEVAGIKSVTIKISGDYAYGWLRTETGVHRLVRKSPFDSGGRRHTSFSSAFVYPEVDD

XS4 RF2      DVSIEINFSDLRIDTYRASGAGGQHVNRTDSAVRVTHLPTNIVVQCQNNRSQHKNKDHAL
RS3 RF2      NIKVEINSSEIRVDTYKSSGAGGQHVNRTESAVRITHEPTSIVVQCQSDRSQHKNKEQAM
E.coli RF2   DIDIEINPADLRIDVYRASGAGGQHVNRTESAVRITHIPTGIVTQCQNDRSQHKNKDQAM

XS4 RF2      SQLKSKLYDLEVKKRNAKKQKLESEKLDITWGNQIRSYILDQSRIKDIRTNIEISNIQSV
RS3 RF2      KQLKSKLYEMKIREKENEKQKENINKSNISWGRHIRSYILDQSRVKDIRTGIENTNPQNV
E.coli RF2   KQMKAKLYELEMQKKNAEKQAMEDNKSDIGWGSQIRSYVLDDSRIKDLRTGVETRNTQAV

XS4 RF2      LDGKIDKFIYAALRL-L
RS3 RF2      LNGNLDEFIEESLKLNL
E.coli RF2   LDGSLDQFIEASLKAGL
```

Supplementary figure S3. Comparison among the RF2 amino acid sequences of *Escherichia coli*, XS4, and RS3. The amino acid residues highlighted in red were observed to interact with UAA/UGA triplet in the crystals of the translation termination complex. Two highly conserved motifs, which are responsible for codon recognition and promoting the hydrolysis of peptidyl-tRNA in the ribosome, are shaded.

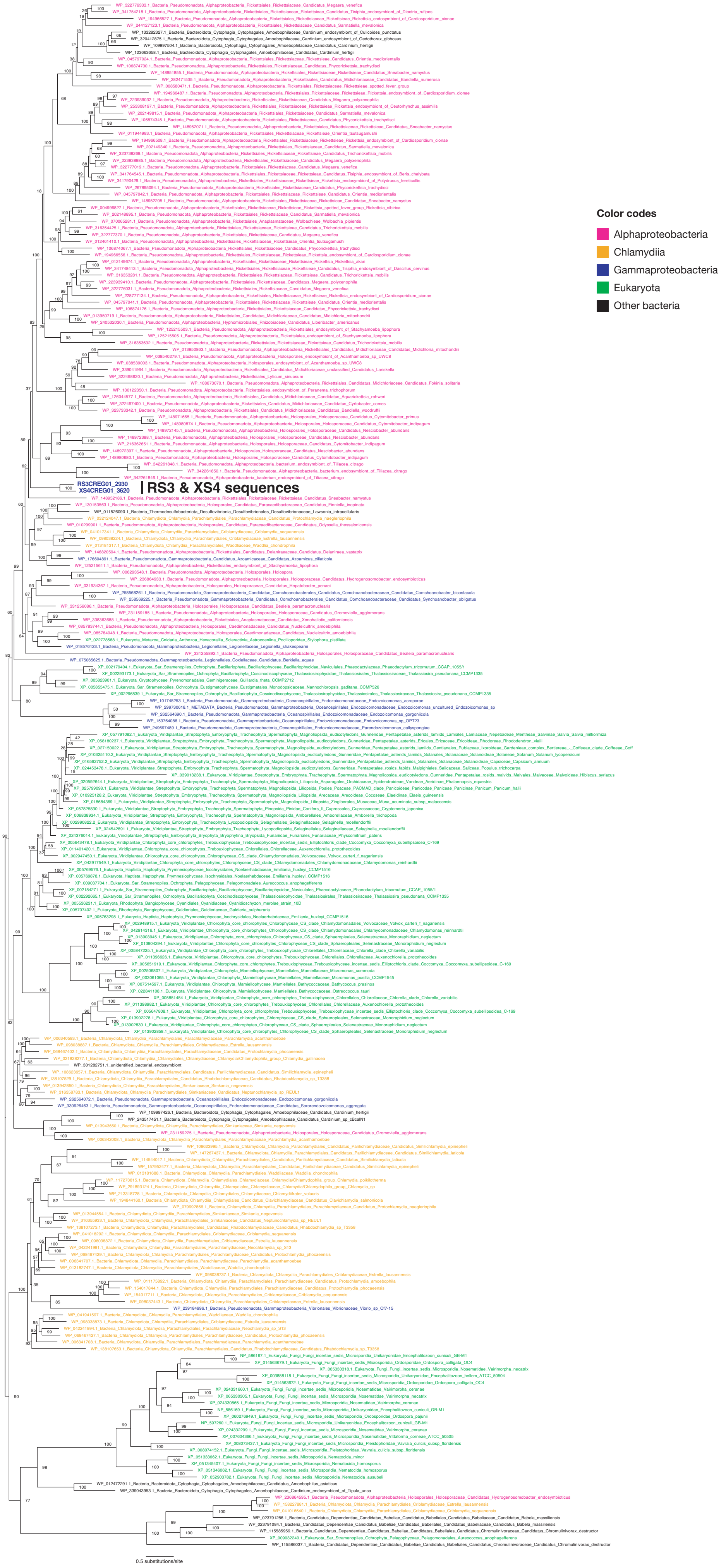

Supplementary figure S4. Maximum likelihood phylogenetic tree based on the ADP:ADP antiporter protein sequence alignment. The tree was inferred using IQ-Tree under the LG+C60+F+G model. The statistical support for each bipartition in the ML tree was calculated by 1000-replicate ultrafast bootstrap approximation. The alignment included 229 taxa and 463 amino acid positions.
